# The PEGASUS Conferences: A unique avenue to bridge evidence and action

**DOI:** 10.7189/jogh.10.010202

**Published:** 2020-06

**Authors:** Michelle Amri, Neil Arya, Steve Ferracuti, Michael Clarke

**Affiliations:** 1 Dalla Lana School of Public Health, University of Toronto, Toronto, Ontario, Canada; 2 International Migration Research Centre, Balsillie School for International Affairs, Waterloo, Ontario, Canada; 3 Wilfrid Laurier University, Waterloo, Ontario, Canada; 4 Haliburton Highlands Health Services, Haliburton, Ontario, Canada; 5 Peterborough Regional Health Centre, Peterborough, Ontario, Canada; 6 Canadian Physicians for Aid and Relief, Toronto, Ontario, Canada; 7 Schulich School of Medicine and Dentistry, Western University, London, Ontario, Canada

The complex and seemingly overwhelming problems our world is facing today require equally complex and, for some, incomprehensible solutions. No single entity – and most definitely, no single individual – is capable of imagining the actions that must be taken to address geopolitical tensions, the climate crisis, economic instability, and general apprehension about the future [1].

As always, recognition and definition of the problems themselves is a necessary first step to resolution. Getting to that point requires collective deliberation followed by consensus-based action. Looking to the Sustainable Development Goals, the consultation process that was globally inclusive [2] is a step in the right direction, but has been critiqued as vague and, while aiming to save the world, does not seem to transform it [3].

Recognition and definition of the problems and evidence-based potential actions do exist for many critical problem areas. There is, however, a human reluctance to pursue the evidence further in order to connect the dots towards truly rooted solutions. An illustration of this is our reluctance at the international, national, and even personal levels to acknowledge that the present capitalist consumptive economic model is not really compatible with climate change salvage or reduction of some economic inequalities.

Global leadership groups such as the G7 may achieve progress and consensus in areas such as trade and policies which promote the growth of capital. Yet these same groups are utterly unable to achieve any meaningful consensus towards areas involving the commons, the sustainable habitation of our planet, income inequality, human rights, and other issues which are emergently pressing. Clearly the process for high level recognition, examination of evidence, prioritization, and meaningful action is flawed, perhaps fatally so.

Almost without exception, global political and business leadership is unable or unwilling to achieve consensus on an action plan that is commensurate with the urgency of these global challenges.

Inspired, informed, selfless, civic leadership is needed to pressure and create the changes that may move the Doomsday clock away from midnight [4]. These movements must be guided by a full understanding of the root causes of these issues (through rigorous research). These movements must aim to progress beyond the local towards the global. This may be guided by evidence-based policies and evidence-based models of change.

Of course, the challenges are great. Those who consistently and vigorously defend the status quo have deep vested interests in industries that extract, market, and sell products that directly threaten global sustainability: in weapons of both personal and mass destruction, in outrageously pricing drugs in the global north and south, from Big Tobacco to Big Oil, to a Military Industrial Complex. These interests are well connected with our elected officials and have deep pockets and their own bodies of research and think tanks which are employed to defend the status quo.

However, even these defenders of the status quo do not recognize the vested interest that they should have to address these issues for their very own children and grandchildren.

As has been seen many times before, public opinion is ahead of effective policy-making [5]. Polls consistently show that global threats to the environment including climate, armed conflict, and health are the top concerns across the entire world [6]. This opinion is broad but may not be deep. As an example, most Canadians support the notion that climate change is at a crisis level but at the very same time are unwilling to even modestly increase their own level of taxation as a means to address this problem [7].

One ongoing exercise that does seek to connect investigations and evidence in the areas of commerce, politics, economics, global health (both locally and internationally), environmental sustainability, and peace is the PEGASUS conference and process. By bringing together students, health practitioners, public health professionals, academics, activists, policy-makers, and public officials from diverse backgrounds involved formally or informally in these areas, the PEGASUS Conferences aims to identify the fundamental issues that are driving the current crises in all three and to bring this knowledge to a broader audience, such as through aiming to influence advocacy and policy making platforms.

The name PEGASUS is inspired to connect the dots (an acronym derived from PEace, Global health And SUStainability). Previous speakers have included activists such as Naomi Klein, peace advocates including Ziauddin Yousefzai, whose daughter Malala reached the Nobel stage, along with other past speakers such as Setsuko Thurlow and James Orbinski, political leaders such as Elizabeth May and Ryan Meili and Indigenous activists from Canada and abroad including Erica Violet Lee and Alfonso Morales.

Hosted last year from April 27 to 29, 2018, at the University of Toronto, the third PEGASUS conference built on the immense wealth of knowledge to extend the platform for meaningful discussion from an impressive array of contributors. In considering how to address the pressing issues confronting contemporary society, PEGASUS III provided a venue to explore the space between thought and action (appropriately titled “from Thought to Action”).

Each of our actions must stem from an idea or thought; one might even add inaction. This is true at the individual level as well as at the organizational level, be it a small non-governmental organization, interest group, academic organization, or government. The space between thought and action is often wide. How do we move from great thought across the divide to meaningful action, whether such action be research, policy, advocacy, activism, or education? Direct or indirect actions can be undertaken rooted in evidence, with the express purpose of changing the status quo.

Given the role of evidence in igniting action, deliberate attention to what constitutes evidence is crucial. A discussion of what constitutes evidence was present in various plenary sessions. James Orbinski opened the conference with a keynote on “Climate Change and Global Health”, Vikram Patel, Charles Larson, Daisy Singla, and Janet Hatcher Roberts led the conference through a discussion on “Scaling Up Effective Interventions” with a session that was aware of the impact of interventions on existing systems and the possible unintended consequences of interventions.

An example of the truly thought-provoking nature of the conference was the workshop session “Decolonizing Evidence”, a critical reflection on what constitutes evidence. This workshop drew on Indigenous ways of knowing and provided a humbling and interesting forum where participants actively discussed how some evidence is given preference over others, resulting in a lack of “cognitive justice”.

Cognitive justice is the idea that all forms of evidence should be equally regarded, but not that all knowledge is equal – also referred to as ‘decolonizing knowledge’ [8]. For example, Western ways of knowing should be treated in a similar way to Indigenous ways of knowing – not that anti-vax knowledge should be treated equally to scientific knowledge. Moving to improved ways of knowing, a suggestion was put forward to employ two-eyed seeing – referring to drawing on Indigenous ways of knowing to see from one eye and Western ways of knowing from the other and using both of these eyes together to see the full picture [9], may allow for the development and acceptance of improved solutions which have the potential to transform through new thinking.

In her workshop titled “Promoting Cohesion in Families and Communities Through the Community Participatory Approach”, Miriam Were from Moi University in Kenya led participants on an exploration of the facets of women’s empowerment and community cohesion which included the thought that in order for women to become fully empowered, men must also become empowered to overcome their actions which may be rooted in self-doubt and lack of self-esteem.

While these insights are being explored throughout the world, explicit acknowledgement is needed that the vision and leadership on these issues will come from: the global south and youth.

In fact, some have opined that in considering knowledge, Africa remains colonized due to a lack of control over data, with particular reference to academic fieldwork [10]. In this domain, the concept of global learning is highly important, and by applying two-eyed seeing globally, all knowledge and evidence is seen as valuable, and credit should be afforded to both local and global knowledge, arising from both the global north and south. A concerted effort must be made to place all knowledge on the playing field. Otherwise, power imbalances can continue to pose challenges in forming partnerships, and ultimately in achieving global goals such as universal health coverage.

We must be open to the fact that “reverse innovation”, evidence that is specifically drawn from the global south, shapes practices in the global north. This includes: medical products (such as a low-cost electrocardiogram (ECG) that was developed to reach rural and low-income areas in India), health information (mapping of eyewitness reports of violence), and service delivery (such as the community health worker model) [11]. However, despite seeking to be a positive approach which recognizes that there are numerous forms of knowledge originating from various places, the term ‘reverse innovation’ does not afford cognitive justice as the term implies that knowledge typically originates in the global north (instead of simply, “innovation”).

The PEGASUS conference illustrates the importance of sharing insights through knowledge translation and exchange and crucially brings in views from both the global north and south. Similarly, the role and importance of youth is highlighted throughout the conferences, with speakers at PEGASUS IV including Dominique Souris, the co-founder and executive director of the Youth Climate Laboratory, and Nishin Nathwani, who is hosting a workshop on the arts with youth. When evidence from various sources are drawn on, and this knowledge is shared, effective policies may be developed. However, it is important to remain cognizant of the policy-making process and understand that evidence is not always drawn on during this process. We must expand our circles and through the open and honest platform of movements such as PEGASUS, play an active role in public policy development. This policy development should also provide justice to other ways of knowing.

This is one of the main tenets of PEGASUS, which is why the conference is built on a foundation of four streams: peace, global health local, global health international, and sustainability. These streams ultimately bring together these unique perspectives to achieve shared goals around improving health for all by advancing awareness and understanding of peace, global health, and sustainability.

PEGASUS clarifies and reminds us that small steps may lead to big change. It is through establishing common ground, strategizing together, and taking small steps, that we are engaging in “re-organized action” – coming together to reorganize by thinking through our preconceptions (such as those around what constitutes evidence) and clarifying next steps for action (whether that be through community-led change or wide-scale global protests, such as those led by Greta Thunberg). Calling attention to the need to discuss, have open lines of communication, and engage in interdisciplinary collaboration is a way to re-organize action. These discussions can center around what works across different contexts and help us to determine what approaches may work and which may not. It is critical that we allow for mutual learning from one another to inform our actions.

It is only then that we can overcome the key challenges of our time – a message of hope in a conflicted world.

Topics examined previously at PEGASUS are cross-cutting by nature. Approaches to research that transcend conventional domains of inquiry are crucial. We invite you to join us in taking a step in this direction.

The next PEGASUS conference, PEGASUS IV, is to be held at the Balsillie School of International Affairs in Waterloo, Canada in spring of 2021. COVID-19 has resulted in postponing the conference and will entail not only reconsidering the format in which it is delivered, but also highlights the need for collective interdisciplinary action involving researchers, educators, policy-makers, and civil society.

As in previous PEGASUS conferences this conference seeks to engage participants in discussions around globally and locally critical problems with a particular focus on: climate change, migration, and sustainable development.

One of the aims is to discuss these issues and their complexity.

For example, are the sustainable development goals (SDGs) justified? Are there inherent flaws with their design? Are we excluding critical aspects through their development? What evidence (or lack thereof) is being used to justify success in this realm? Are the SDGs applicable to local contexts? These, among various other pertinent questions will be raised in the hopes of re-organizing action.

We will be joined by numerous experts, ranging from: individuals in municipal government (Dr Cyrille Kiyungu, the Mayor of Kikwit, Congo during the 1995 Ebola outbreak), federal government (Dr Patricia Garcia, former Minister of Health in Peru), those with multilateral organizations (Rosemary McCarney, formerly Canada's Ambassador to the United nations in Geneva and head of Plan Canada), researchers and knowledge creators (such as Professor Sir Andy Haines, an expert on environmental influences on health; Dr Jeffrey Sachs, economist, professor, and central player in the development the SDGs; Dr Corinne Schuster-Wallace who studies climate change; and Dr Ingrid Waldron of Dalhousie University who studies equity and racism) and the President of the Canadian Medical Association, Dr Sandy Buchman who also works in palliative care and homeless individuals in the inner city.

We hope you will join us at the next PEGASUS conference (www.pegasusconference.ca) to engage in critical, disruptive, visionary, visible, respected, and effective conversations and mobilize action, so that we can come one step closer to achieving a peaceful, sustainable, and healthy world for all.
